# Supplementary material for: In vitro detection of canine anti-human antibodies following intratumoral injection of the hu14.18-IL2 immunocytokine in spontaneous canine melanoma
Source: bioRxiv. 2025 Mar 27:2025.03.21.644578. Preprint. [Version 2] doi: 10.1101/2025.03.21.644578 (PMC11974718; doi:10.1101/2025.03.21.644578)
Supplement: Supplement 1 [file media-1.pdf]

arm	patient.id	tmpt	MFI
A	ITIC-04	Baseline	10602
A	ITIC-04	Baseline	12184
A	ITIC-04	Baseline	13544
A	ITIC-04	Day 1	13327
A	ITIC-04	Day 1	13023
A	ITIC-04	Day 1	12757
A	ITIC-04	Day 10	1145
A	ITIC-04	Day 10	1026
A	ITIC-04	Day 10	2076
A	ITIC-04	Day 30	195
A	ITIC-04	Day 30	191
A	ITIC-04	Day 30	265
A	ITIC-04	Day 60	5393
A	ITIC-04	Day 60	4997
A	ITIC-04	Day 60	5147
B	ITIC-05	Baseline	10459
B	ITIC-05	Baseline	10578
B	ITIC-05	Baseline	11020
B	ITIC-05	Day 1	10110
B	ITIC-05	Day 1	11045
B	ITIC-05	Day 1	11377
B	ITIC-05	Day 10	727
B	ITIC-05	Day 10	722
B	ITIC-05	Day 10	873
B	ITIC-05	Day 30	204
B	ITIC-05	Day 30	121
B	ITIC-05	Day 30	112
B	ITIC-05	Day 60	146
B	ITIC-05	Day 60	118
B	ITIC-05	Day 60	123
A	ITIC-06	Baseline	19576
A	ITIC-06	Baseline	18941
A	ITIC-06	Baseline	18200
A	ITIC-06	Day 1	19995
A	ITIC-06	Day 1	20184
A	ITIC-06	Day 1	18545
A	ITIC-06	Day 10	4701
A	ITIC-06	Day 10	5180
A	ITIC-06	Day 10	4751

A	ITIC-06	Day 30	516
A	ITIC-06	Day 30	470
A	ITIC-06	Day 30	561
B	ITIC-07	Baseline	18633
B	ITIC-07	Baseline	17572
B	ITIC-07	Baseline	16382
B	ITIC-07	Day 1	17654
B	ITIC-07	Day 1	18808
B	ITIC-07	Day 1	19075
B	ITIC-07	Day 10	14584
B	ITIC-07	Day 10	15746
B	ITIC-07	Day 10	15672
B	ITIC-07	Day 30	1950
B	ITIC-07	Day 30	1237
B	ITIC-07	Day 30	1397
B	ITIC-07	Day 60	11507
B	ITIC-07	Day 60	9534
B	ITIC-07	Day 60	10202
B	ITIC-08	Baseline	18676
B	ITIC-08	Baseline	18415
B	ITIC-08	Baseline	16730
B	ITIC-08	Day 1	16268
B	ITIC-08	Day 1	18115
B	ITIC-08	Day 1	19576
B	ITIC-08	Day 10	13388
B	ITIC-08	Day 10	12815
B	ITIC-08	Day 10	13388
B	ITIC-08	Day 30	614
B	ITIC-08	Day 30	725
B	ITIC-08	Day 30	599
B	ITIC-08	Day 60	2410
B	ITIC-08	Day 60	1680
B	ITIC-08	Day 60	1335
A	ITIC-09	Baseline	17246
A	ITIC-09	Baseline	18502
A	ITIC-09	Baseline	20184
A	ITIC-09	Day 1	21060
A	ITIC-09	Day 1	23538
A	ITIC-09	Day 1	23206
A	ITIC-09	Day 10	1394

A	ITIC-09	Day 10	1195
A	ITIC-09	Day 10	1136
A	ITIC-09	Day 30	622
A	ITIC-09	Day 30	828
A	ITIC-09	Day 60	487
A	ITIC-09	Day 60	603
A	ITIC-09	Day 60	568
A	ITIC-10	Baseline	20666
A	ITIC-10	Baseline	21975
A	ITIC-10	Baseline	20911
A	ITIC-10	Day 1	18243
A	ITIC-10	Day 1	17006
A	ITIC-10	Day 1	17327
A	ITIC-10	Day 10	14151
A	ITIC-10	Day 10	12296
A	ITIC-10	Day 10	14151
A	ITIC-10	Day 30	1983
A	ITIC-10	Day 30	1950
A	ITIC-10	Day 30	1518
A	ITIC-10	Day 60	10578
A	ITIC-10	Day 60	9773
A	ITIC-10	Day 60	9364
B	ITIC-11	Baseline	12073
B	ITIC-11	Baseline	14054
B	ITIC-11	Baseline	13701
B	ITIC-11	Day 1	13956
B	ITIC-11	Day 1	14857
B	ITIC-11	Day 1	14891
B	ITIC-11	Day 10	9951
B	ITIC-11	Day 10	9074
B	ITIC-11	Day 10	11586
B	ITIC-11	Day 30	7414
B	ITIC-11	Day 30	7284
B	ITIC-11	Day 30	6377
B	ITIC-11	Day 60	7612
B	ITIC-11	Day 60	9664
B	ITIC-11	Day 60	8993
A	ITIC-12	Baseline	12727
A	ITIC-12	Baseline	13956
A	ITIC-12	Baseline	14151

A	ITIC-12	Day 1	14961
A	ITIC-12	Day 1	14151
A	ITIC-12	Day 1	15967
A	ITIC-12	Day 10	8448
A	ITIC-12	Day 10	9197
A	ITIC-12	Day 10	8081
A	ITIC-12	Day 30	1145
A	ITIC-12	Day 30	1278
A	ITIC-12	Day 30	1246
A	ITIC-13	Baseline	12325
A	ITIC-13	Baseline	13956
A	ITIC-13	Baseline	13357
A	ITIC-13	Day 1	14754
A	ITIC-13	Day 1	13419
A	ITIC-13	Day 1	15819
A	ITIC-13	Day 10	15856
A	ITIC-13	Day 10	17531
A	ITIC-13	Day 10	15455
A	ITIC-13	Day 30	10723
A	ITIC-13	Day 30	14054
A	ITIC-13	Day 30	15100
A	ITIC-13	Day 60	13204
A	ITIC-13	Day 60	15030
A	ITIC-13	Day 60	14151
B	ITIC-14	Baseline	15312
B	ITIC-14	Baseline	14857
B	ITIC-14	Baseline	16887
B	ITIC-14	Day 1	16966
B	ITIC-14	Day 1	18720
B	ITIC-14	Day 1	16420
B	ITIC-14	Day 10	13796
B	ITIC-14	Day 10	13481
B	ITIC-14	Day 10	13606
B	ITIC-14	Day 30	13388
B	ITIC-14	Day 30	14483
B	ITIC-14	Day 30	14382
B	ITIC-14	Day 60	13481
B	ITIC-14	Day 60	11693
B	ITIC-14	Day 60	14995
B	ITIC-15	Baseline	16004

B	ITIC-15	Baseline	17696
B	ITIC-15	Baseline	16497
B	ITIC-15	Day 1	15065
B	ITIC-15	Day 1	14021
B	ITIC-15	Day 1	14857
B	ITIC-15	Day 10	12496
B	ITIC-15	Day 10	13512
B	ITIC-15	Day 10	13701
B	ITIC-15	Day 30	3070
B	ITIC-15	Day 30	2095
B	ITIC-15	Day 30	1931
B	ITIC-15	Day 60	4116
B	ITIC-15	Day 60	4246
B	ITIC-15	Day 60	3761

arm	patient.id	tmpt	OD
A	ITIC-04	Neg Control	0.169
A	ITIC-04	Neg Control	0.123
A	ITIC-04	Neg Control	0.125
A	ITIC-04	Neg Control	0.155
A	ITIC-04	Baseline	0.174
A	ITIC-04	Baseline	0.183
A	ITIC-04	Baseline	0.171
A	ITIC-04	Baseline	0.142
A	ITIC-04	Day 1	0.185
A	ITIC-04	Day 1	0.174
A	ITIC-04	Day 1	0.099
A	ITIC-04	Day 1	0.217
A	ITIC-04	Day 10	0.191
A	ITIC-04	Day 10	0.162
A	ITIC-04	Day 10	0.191
A	ITIC-04	Day 10	0.145
A	ITIC-04	Day 30	0.213
A	ITIC-04	Day 30	0.206
A	ITIC-04	Day 30	0.226
A	ITIC-04	Day 30	0.238
A	ITIC-04	Day 60	0.287
A	ITIC-04	Day 60	0.222
A	ITIC-04	Day 60	0.163
A	ITIC-04	Day 60	0.186
B	ITIC-05	Neg Control	0.148
B	ITIC-05	Neg Control	0.148
B	ITIC-05	Neg Control	0.132
B	ITIC-05	Neg Control	0.124
B	ITIC-05	Baseline	0.116
B	ITIC-05	Baseline	0.137
B	ITIC-05	Baseline	0.153
B	ITIC-05	Baseline	0.148
B	ITIC-05	Day 1	0.135
B	ITIC-05	Day 1	0.155
B	ITIC-05	Day 1	0.142
B	ITIC-05	Day 1	0.172
B	ITIC-05	Day 10	0.167
B	ITIC-05	Day 10	0.233
B	ITIC-05	Day 10	0.248

B	ITIC-05	Day 10	0.162
B	ITIC-05	Day 30	0.250
B	ITIC-05	Day 30	0.194
B	ITIC-05	Day 30	0.300
B	ITIC-05	Day 30	0.268
B	ITIC-05	Day 60	0.332
B	ITIC-05	Day 60	0.199
B	ITIC-05	Day 60	0.221
B	ITIC-05	Day 60	0.295
A	ITIC-06	Neg Control	0.148
A	ITIC-06	Neg Control	0.148
A	ITIC-06	Neg Control	0.132
A	ITIC-06	Neg Control	0.124
A	ITIC-06	Baseline	0.176
A	ITIC-06	Baseline	0.127
A	ITIC-06	Baseline	0.112
A	ITIC-06	Baseline	0.115
A	ITIC-06	Day 1	0.112
A	ITIC-06	Day 1	0.176
A	ITIC-06	Day 1	0.191
A	ITIC-06	Day 1	0.145
A	ITIC-06	Day 10	0.148
A	ITIC-06	Day 10	0.153
A	ITIC-06	Day 10	0.206
A	ITIC-06	Day 10	0.119
A	ITIC-06	Day 30	0.253
A	ITIC-06	Day 30	0.286
A	ITIC-06	Day 30	0.230
A	ITIC-06	Day 30	0.208
B	ITIC-07	Neg Control	0.237
B	ITIC-07	Neg Control	0.243
B	ITIC-07	Neg Control	0.165
B	ITIC-07	Neg Control	0.228
B	ITIC-07	Baseline	0.254
B	ITIC-07	Baseline	0.165
B	ITIC-07	Baseline	0.208
B	ITIC-07	Baseline	0.197
B	ITIC-07	Day 1	0.318
B	ITIC-07	Day 1	0.244
B	ITIC-07	Day 1	0.181

B	ITIC-07	Day 1	0.138
B	ITIC-07	Day 10	0.261
B	ITIC-07	Day 10	0.229
B	ITIC-07	Day 10	0.224
B	ITIC-07	Day 10	0.184
B	ITIC-07	Day 30	0.213
B	ITIC-07	Day 30	0.254
B	ITIC-07	Day 30	0.272
B	ITIC-07	Day 30	0.366
B	ITIC-07	Day 60	0.275
B	ITIC-07	Day 60	0.393
B	ITIC-07	Day 60	0.274
B	ITIC-07	Day 60	0.266
B	ITIC-08	Neg Control	0.105
B	ITIC-08	Neg Control	0.089
B	ITIC-08	Neg Control	0.087
B	ITIC-08	Neg Control	0.110
B	ITIC-08	Baseline	0.113
B	ITIC-08	Baseline	0.139
B	ITIC-08	Baseline	0.126
B	ITIC-08	Baseline	0.113
B	ITIC-08	Day 1	0.120
B	ITIC-08	Day 1	0.126
B	ITIC-08	Day 1	0.127
B	ITIC-08	Day 1	0.116
B	ITIC-08	Day 10	0.122
B	ITIC-08	Day 10	0.130
B	ITIC-08	Day 10	0.134
B	ITIC-08	Day 10	0.130
B	ITIC-08	Day 30	0.124
B	ITIC-08	Day 30	0.150
B	ITIC-08	Day 30	0.147
B	ITIC-08	Day 30	0.144
B	ITIC-08	Day 60	0.166
B	ITIC-08	Day 60	0.160
B	ITIC-08	Day 60	0.142
B	ITIC-08	Day 60	0.141
A	ITIC-09	Neg Control	0.105
A	ITIC-09	Neg Control	0.099
A	ITIC-09	Neg Control	0.097

A	ITIC-09	Neg Control	0.120
A	ITIC-09	Baseline	0.115
A	ITIC-09	Baseline	0.118
A	ITIC-09	Baseline	0.114
A	ITIC-09	Baseline	0.119
A	ITIC-09	Day 1	0.153
A	ITIC-09	Day 1	0.094
A	ITIC-09	Day 1	0.106
A	ITIC-09	Day 1	0.118
A	ITIC-09	Day 10	0.129
A	ITIC-09	Day 10	0.118
A	ITIC-09	Day 10	0.096
A	ITIC-09	Day 10	0.114
A	ITIC-09	Day 30	0.181
A	ITIC-09	Day 30	0.208
A	ITIC-09	Day 30	0.158
A	ITIC-09	Day 30	0.164
A	ITIC-09	Day 60	0.157
A	ITIC-09	Day 60	0.162
A	ITIC-09	Day 60	0.127
A	ITIC-09	Day 60	0.139
A	ITIC-10	Neg Control	0.105
A	ITIC-10	Neg Control	0.089
A	ITIC-10	Neg Control	0.087
A	ITIC-10	Neg Control	0.110
A	ITIC-10	Baseline	0.139
A	ITIC-10	Baseline	0.130
A	ITIC-10	Baseline	0.089
A	ITIC-10	Baseline	0.140
A	ITIC-10	Day 1	0.114
A	ITIC-10	Day 1	0.159
A	ITIC-10	Day 1	0.091
A	ITIC-10	Day 1	0.101
A	ITIC-10	Day 10	0.126
A	ITIC-10	Day 10	0.116
A	ITIC-10	Day 10	0.098
A	ITIC-10	Day 10	0.092
A	ITIC-10	Day 30	0.128
A	ITIC-10	Day 30	0.103
A	ITIC-10	Day 30	0.124

A	ITIC-10	Day 60	0.139
A	ITIC-10	Day 60	0.122
A	ITIC-10	Day 60	0.106
B	ITIC-11	Neg Control	0.237
B	ITIC-11	Neg Control	0.243
B	ITIC-11	Neg Control	0.165
B	ITIC-11	Neg Control	0.228
B	ITIC-11	Baseline	0.218
B	ITIC-11	Baseline	0.270
B	ITIC-11	Baseline	0.257
B	ITIC-11	Baseline	0.204
B	ITIC-11	Day 1	0.179
B	ITIC-11	Day 1	0.261
B	ITIC-11	Day 1	0.274
B	ITIC-11	Day 1	0.217
B	ITIC-11	Day 10	0.242
B	ITIC-11	Day 10	0.250
B	ITIC-11	Day 10	0.240
B	ITIC-11	Day 10	0.162
B	ITIC-11	Day 30	0.338
B	ITIC-11	Day 30	0.413
B	ITIC-11	Day 30	0.314
B	ITIC-11	Day 30	0.342
B	ITIC-11	Day 60	0.292
B	ITIC-11	Day 60	0.354
B	ITIC-11	Day 60	0.329
B	ITIC-11	Day 60	0.316
A	ITIC-12	Neg Control	0.237
A	ITIC-12	Neg Control	0.243
A	ITIC-12	Neg Control	0.165
A	ITIC-12	Neg Control	0.228
A	ITIC-12	Baseline	0.257
A	ITIC-12	Baseline	0.234
A	ITIC-12	Baseline	0.234
A	ITIC-12	Baseline	0.172
A	ITIC-12	Day 1	0.272
A	ITIC-12	Day 1	0.259
A	ITIC-12	Day 1	0.157
A	ITIC-12	Day 1	0.177
A	ITIC-12	Day 10	0.246

A	ITIC-12	Day 10	0.159
A	ITIC-12	Day 10	0.151
A	ITIC-12	Day 10	0.204
A	ITIC-12	Day 30	0.258
A	ITIC-12	Day 30	0.323
A	ITIC-12	Day 30	0.267
A	ITIC-12	Day 30	0.323
A	ITIC-13	Neg Control	0.159
A	ITIC-13	Neg Control	0.129
A	ITIC-13	Neg Control	0.123
A	ITIC-13	Neg Control	0.090
A	ITIC-13	Baseline	0.113
A	ITIC-13	Baseline	0.183
A	ITIC-13	Baseline	0.138
A	ITIC-13	Baseline	0.194
A	ITIC-13	Day 1	0.122
A	ITIC-13	Day 1	0.174
A	ITIC-13	Day 1	0.131
A	ITIC-13	Day 1	0.134
A	ITIC-13	Day 10	0.133
A	ITIC-13	Day 10	0.135
A	ITIC-13	Day 10	0.141
A	ITIC-13	Day 10	0.192
A	ITIC-13	Day 30	0.139
A	ITIC-13	Day 30	0.129
A	ITIC-13	Day 30	0.171
A	ITIC-13	Day 30	0.144
A	ITIC-13	Day 60	0.141
A	ITIC-13	Day 60	0.172
A	ITIC-13	Day 60	0.156
A	ITIC-13	Day 60	0.181
B	ITIC-14	Neg Control	0.159
B	ITIC-14	Neg Control	0.129
B	ITIC-14	Neg Control	0.123
B	ITIC-14	Neg Control	0.090
B	ITIC-14	Baseline	0.164
B	ITIC-14	Baseline	0.134
B	ITIC-14	Baseline	0.175
B	ITIC-14	Baseline	0.165
B	ITIC-14	Day 1	0.169

B	ITIC-14	Day 1	0.166
B	ITIC-14	Day 1	0.191
B	ITIC-14	Day 1	0.143
B	ITIC-14	Day 10	0.106
B	ITIC-14	Day 10	0.128
B	ITIC-14	Day 10	0.208
B	ITIC-14	Day 10	0.228
B	ITIC-14	Day 30	0.444
B	ITIC-14	Day 30	0.198
B	ITIC-14	Day 30	0.258
B	ITIC-14	Day 30	0.238
B	ITIC-14	Day 60	0.152
B	ITIC-14	Day 60	0.225
B	ITIC-14	Day 60	0.195
B	ITIC-14	Day 60	0.235
B	ITIC-15	Neg Control	0.129
B	ITIC-15	Neg Control	0.123
B	ITIC-15	Neg Control	0.090
B	ITIC-15	Neg Control	0.125
B	ITIC-15	Baseline	0.154
B	ITIC-15	Baseline	0.164
B	ITIC-15	Baseline	0.128
B	ITIC-15	Baseline	0.132
B	ITIC-15	Day 1	0.168
B	ITIC-15	Day 1	0.167
B	ITIC-15	Day 1	0.199
B	ITIC-15	Day 1	0.126
B	ITIC-15	Day 10	0.211
B	ITIC-15	Day 10	0.204
B	ITIC-15	Day 10	0.169
B	ITIC-15	Day 10	0.155
B	ITIC-15	Day 30	0.189
B	ITIC-15	Day 30	0.206
B	ITIC-15	Day 30	0.237
B	ITIC-15	Day 30	0.186
B	ITIC-15	Day 60	0.201
B	ITIC-15	Day 60	0.199
B	ITIC-15	Day 60	0.180
B	ITIC-15	Day 60	0.187
