# Supplementary material for: In vitro detection of canine anti-human antibodies following intratumoral injection of the hu14.18-IL2 immunocytokine in spontaneous canine melanoma
Source: bioRxiv. 2025 Mar 27:2025.03.21.644578. Preprint. [Version 2] doi: 10.1101/2025.03.21.644578 (PMC11974718; doi:10.1101/2025.03.21.644578)
Supplement: Supplement 2 [file NIHPP2025.03.21.644578v2-supplement-2.pdf]

bioRxiv preprint doi: https://doi.org/10.1101/2025.03.21.644578; this version posted March 27, 2025. The copyright holder for this preprint (which was not certified by peer review) is the author/funder. This article is a US Government work. It is not subject to copyright under 17 USC 105 and is also made available for use under a CC0 license.

Supporting information

S1 Fig. Binding inhibition assay controls. PE-conjugated anti-GD2 antibody 14G2a was mixed with either (A) 20 μl (open histogram with solid line) or (B) 2.5 μl (open histogram with solid line) healthy canine donor sera before staining M21 cells. M21 stained with 14G2a-PE mixed with 20 μl or 2.5 μl PBS as a no-serum control is represented as a gray histogram in both (A) and (B). Data are representative of triplicates. Values represent % binding inhibition compared to no-serum control.

S2 Fig. Serum from dogs treated with hu14.18-IL2 shows varied level of inhibition of 14G2a binding to target GD2. Prior to staining M21 cells, PE-conjugated anti-GD2 antibody 14G2a was mixed with baseline/pre-treatment sera or post-treatment sera from different timepoints from (A) K914 or (B) K915. Baseline/pre-treatment data are represented as gray histograms; post-treatment data are represented as open histograms with solid lines. Data are representative of triplicates. Values represent % binding inhibition compared to baseline/pre-treatment control.

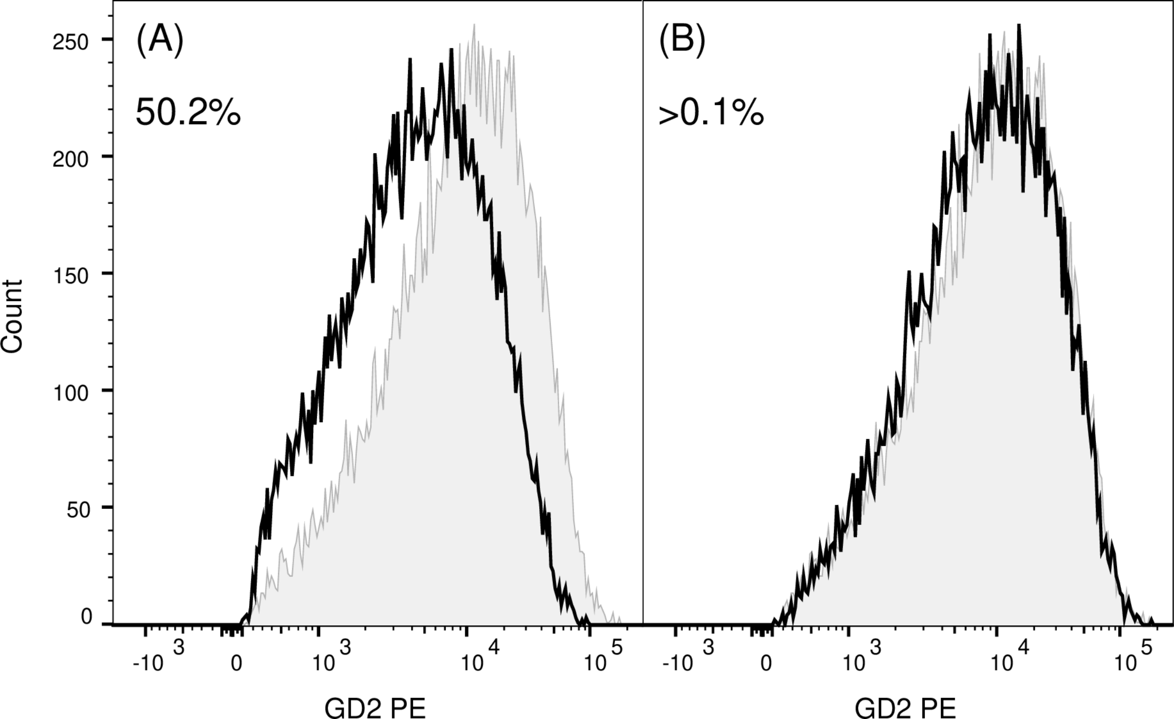

(A)
50.2%
(B)
>0.1%
Count
250
200
150
100
50
0
-10 3
0
10 3
10 4
10 5
GD2 PE
GD2 PE

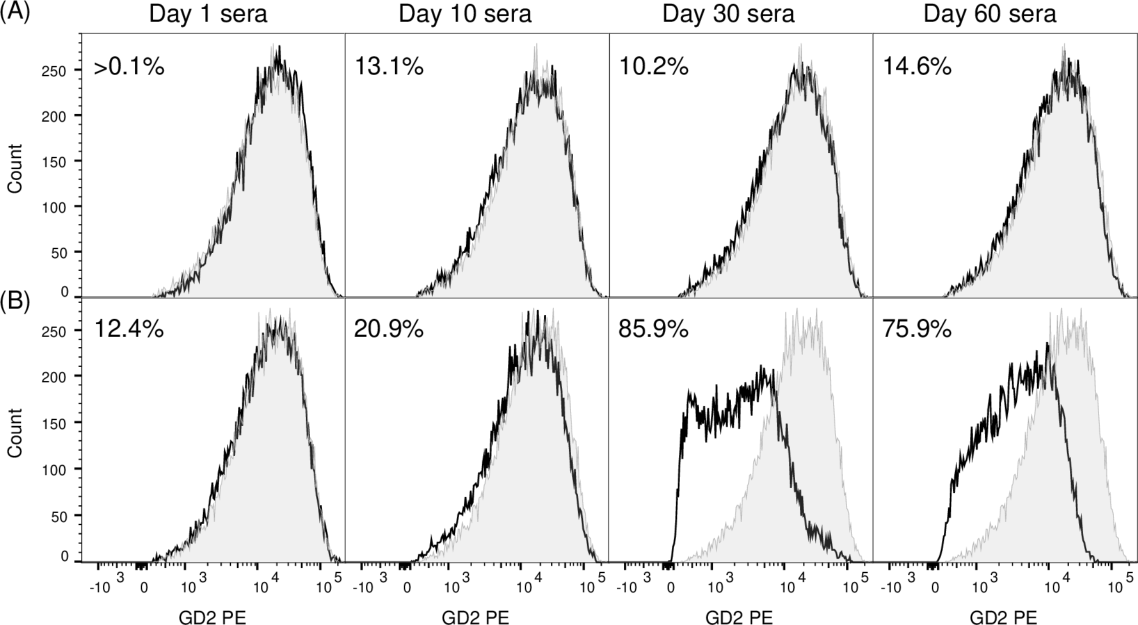

(A)
Day 1 sera
Day 10 sera
Day 30 sera
Day 60 sera
>0.1%
13.1%
10.2%
14.6%
(B)
12.4%
20.9%
85.9%
75.9%
Count
0
50
100
150
200
250
GD2 PE
-10 3
0
10 3
10 4
10 5
